# Cellular Cofactors of Lentiviral Integrase: From Target Validation to Drug Discovery

**DOI:** 10.1155/2012/863405

**Published:** 2012-08-07

**Authors:** Oliver Taltynov, Belete A. Desimmie, Jonas Demeulemeester, Frauke Christ, Zeger Debyser

**Affiliations:** The Laboratory for Molecular Virology and Gene Therapy, KU Leuven, Leuven, Flanders, Belgium

## Abstract

To accomplish their life cycle, lentiviruses make use of host proteins, the so-called cellular cofactors. Interactions between host cell and viral proteins during early stages of lentiviral infection provide attractive new antiviral targets. The insertion of lentiviral cDNA in a host cell chromosome is a step of no return in the replication cycle, after which the host cell becomes a permanent carrier of the viral genome and a producer of lentiviral progeny. Integration is carried out by integrase (IN), an enzyme playing also an important role during nuclear import. Plenty of cellular cofactors of HIV-1 IN have been proposed. To date, the lens epithelium-derived growth factor (LEDGF/p75) is the best studied cofactor of HIV-1 IN. Moreover, small molecules that block the LEDGF/p75-IN interaction have recently been developed for the treatment of HIV infection. The nuclear import factor transportin-SR2 (TRN-SR2) has been proposed as another interactor of HIV IN-mediating nuclear import of the virus. Using both proteins as examples, we will describe approaches to be taken to identify and validate novel cofactors as new antiviral targets. Finally, we will highlight recent advances in the design and the development of small-molecule inhibitors binding to the LEDGF/p75-binding pocket in IN (LEDGINs).

## 1. Introduction: Cofactors of Integration as Potential Antiviral Targets

Infection with the human immunodeficiency virus type 1 (HIV-1) remains a substantial public health as well as a socioeconomic problem worldwide [1]. Although highly active antiretroviral therapy (HAART) effectively halts HIV replication and profoundly increases survival of patients, it has not been possible yet to achieve a cure. Interruption of HAART typically results in a rebound of virus replication. This is primarily due to the fact that HIV has evolved mechanisms to escape from the continuous immune surveillance in a small pool of latently infected cells that are not susceptible to drug therapy. These latently infected cells reside in reservoirs where the distribution of antiretroviral (ARV) drugs is extremely variable and often lower than the expected maximal inhibitory concentration (for recent reviews see [2–4]). Moreover, the rapid replication rate and the generation of an extensive genetic diversity fuel the emergence of drug-resistant viral strains resulting in treatment failure [5, 6]. Therefore, there is a continuous demand to search for novel and better ARVs for a better control of the HIV pandemic with the hope to eventually induce permanent remission of the disease. 

HIV relies on the host cellular machinery to complete its replication cycle. HIV hijacks several biological processes and protein complexes of the host cell through distinct virus-host protein-protein interactions (PPIs) [7, 8]. Since these host-pathogen interactions directly mediate viral replication and disease progression, their specific disruption can provide alternative targets for therapeutic intervention. PPIs represent an attractive group of biologically relevant targets for the development of small-molecule protein-protein interaction inhibitors (SMIPPIs) [9–11]. Since protein-protein interfaces are often based on extended, flat, barely defined, and large hydrophobic surfaces, overcoming binding energy with small molecules is hard to achieve. Therefore, obtaining validated starting points for chemical optimization of SMIPPIs has been difficult [11]. Moreover, the applicability of PPIs as therapeutic targets is not only defined by their physicochemical properties but also by the biological properties of the protein-protein interaction and requires meticulous target validation prior to drug discovery. 

In recent years, our understanding of the HIV-host interaction has dramatically increased, opening the possibility for the discovery of novel classes of therapeutics [8, 12–14]. Not surprisingly, there are numerous interactions between HIV and cellular proteins involved in all stages of virus replication [8]. In principle, any distinct interaction between virus-encoded proteins and host cofactors has the potential to be a target for drug design. The CCR5 antagonist, maraviroc, was approved as the first ARV targeting a host factor [15]. Maraviroc binds to the CCR5 coreceptor on the surface of cells and prevents interaction with the gp120 envelope protein of the virus [16]. Successful targeting of host-virus PPIs demonstrates that HIV-1 therapeutic drug targets are not limited to virus-encoded enzymes and that understanding of the virus-host interactome can be the basis for future HIV therapeutics [17–20]. In theory, this antiviral strategy is expected to make it more difficult for the virus to develop resistance. Since the host factor is genetically conserved in a biologically relevant host-virus interaction, resistance is less likely to occur increasing the clinical potential of these drugs. Alternatively, drug-induced mutations at a conserved interface may reduce viral fitness [21].

**Figure 1:**
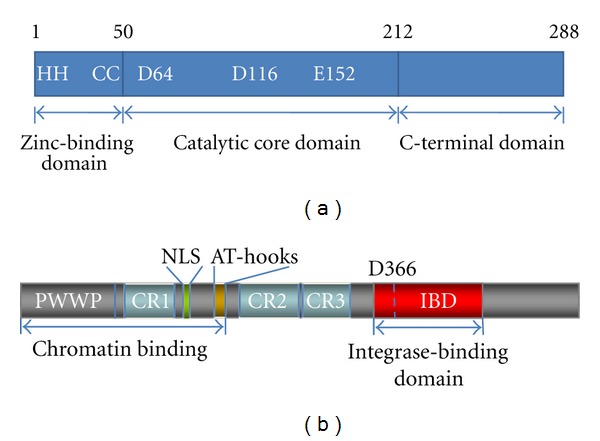
Domain organization of HIV-1 IN and LEDGF/p75. (a) HIV-1 IN is composed of an N-terminal domain (NTD), a catalytic core domain (CCD), and a C-terminal domain (CTD). The CCD contains the catalytically essential DD(35)E motif and the hot spots for interaction with the IBD in LEDGF/p75. The Asp and Glu residues of the CCD coordinate one or two Mg^2+^ ions and are involved in 3′ processing and DNA strand-transfer activities. (b) LEDGF/p75 has several structural motifs involved in chromatin tethering and protein-protein interactions. The PWWP domain, the charged regions (CRs), and AT-hooks are involved in chromatin binding. The C-terminus contains the well-characterized IN binding domain (IBD) and acts as a protein interaction playground. Asp residue 366 critical for HIV-1 IN binding is indicated.

In recent years, HIV-1 integrase (IN) joined the selection of important therapeutic targets to treat HIV infection (for a review see [22]). The enzyme orchestrates the insertion of the viral DNA into the host chromatin [23, 24]. HIV IN is a 32-kDa protein containing 3 canonical structural domains connected by flexible linkers: the N-terminal (NTD, residues 1–50), the catalytic core (CCD, residues 51–212), and the C-terminal domain (CTD, 213–270) (Figure 1(a)). All 3 domains are required for 3′ processing and DNA strand transfer. The solution structure of the N-terminal HHCC domain revealed a three-helix bundle stabilized by zinc [25]. The central catalytic core domain contains the DD(35)E motif conserved among retroviruses and retrotransposons. D64, D116, E152 residues coordinate 2 Mg^2+^ ions necessary for catalysis [26]. The C-terminal domain has a SH3-like fold [27]. Full-length HIV-1 IN is a multimeric enzyme and forms stable tetramers in solution [28].

Despite the recent release of the crystal structure of full-length IN of the prototype foamy virus (PFV) [29], we still lack a crystal structure of full-length HIV-1 IN. The main obstacle for structural studies of HIV IN is its propensity to aggregate. The published two-domain crystal structures of HIV-1 IN (comprising the N-terminal and the catalytic core or the catalytic core and the C-terminal domain) [30, 31] as well as the crystal and NMR structures of individual domains (for review see [32]) represent valuable, but incomplete information on the functional structure of the HIV intasome. HIV integrase was the last HIV enzyme to be effectively targeted with small molecules. Reasons were the lack of homologous disease targets, as opposed to well-studied DNA polymerases and aspartyl proteases and the absence of a crystal structure. Indeed, nowadays structural information is playing a central role in successful drug development. HIV protease (PR) was already recognized as a target in the early nineties [33], and soon after the first crystal structure of HIV-1 PR was published [34]. Publication of the structure of HIV-1 PR complexed with the inhibitor MVT-101 preceded only by six years the approval of the first PR inhibitor as an anti-HIV drug [35, 36].

After completion of reverse transcription, the so-called preintegration complex (PIC) is formed. Along with viral cDNA and IN, the PIC contains viral reverse transcriptase (RT), nucleocapsid (NC), matrix (MA), and Vpr. RT and NC are involved in the synthesis of viral cDNA, while MA and Vpr may affect nuclear import of the PIC. The PIC also contains host cell proteins, and nuclear import is mediated by the interaction with transport factors and nucleoporins. In the nucleus, HIV IN catalyzes the stable insertion of the viral cDNA into a host chromosome.

The recent success in the application of structure-based rational drug design in the discovery and development of allosteric HIV-1 integrase (IN) inhibitors, the LEDGINs [37], was possible due to 7 years of intensive basic research on the cofactor lens epithelium-derived growth factor/p75 (LEDGF/p75). LEDGINs inhibit the interaction between LEDGF/p75 and HIV-1 IN and will be used as an example to discuss approaches, challenges, and future perspectives of SMIPPIIs. 

## 2. Identification and Validation of Cofactors as Novel Antiviral Targets

Purified proteins from diverse sources could rescue the intermolecular integration activity of retroviral PICs isolated from infected cells and salt-stripped of associated host factors. This observation opened a new field in retrovirology focused on the so-called cellular cofactors of retroviral integration (for review see [38]). Farnet and Bushman noticed that a factor important for integration activity *in vitro* was removed upon gel filtration of HIV-1 PICs in the presence of high salt [39]. The activity could be restored by addition of protein extracts from uninfected human SupT1 cells. The factor was identified as the high-mobility-group chromosomal protein A1 (HMGA1, HMG I(Y) protein) [39]. HMGA1 is a nonhistone DNA-binding protein involved in the regulation of inducible gene transcription and microRNA expression [40] in both benign and malignant neoplasias [41]. The same method led to discovery of another cellular cofactor of HIV, barrier-to-autointegration factor (BAF) [42]. By combining antibodies against known viral and cellular PIC components (MA, Vpr, Ku-80) with anti-BAF antibodies, Lin and Engelman proved that human BAF is a component of PIC [43]. Their functional coimmunoprecipitation strategy was based on examining different fractions obtained from HIV-1-infected C8166 T-cells for the presence of integration activity, viral IN and endogenous BAF [43]. Although BAF was suggested to protect retroviral DNA from autointegration and also to promote the association of PICs with target DNA [44], knockdown of BAF by siRNA in HeLaP4 cells did not affect HIV-1 replication [45]. Validation of the role of cellular cofactors in lentiviral infection, thus, requires multiple independent experimental approaches. 

**Figure 2:**
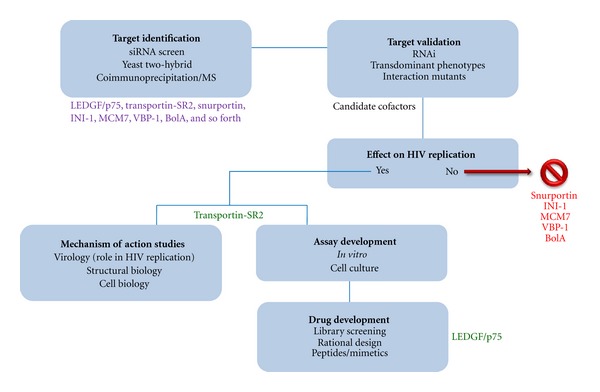
Algorithm to identify and validate novel cofactors as antiviral targets with examples of candidate and validated HIV-1 IN cellular cofactors at particular stages of validation. The algorithm was used in the validation of LEDGF/p75 and TRN-SR2 as cellular cofactors of HIV-1 IN and in validating LEDGF/p75 as an antiviral target. In case of some candidate cofactors, the experimental intervention verifying affect on HIV replication was accompanied by toxicity. These candidates were excluded from follow-up steps of drug target validation.These proteins can still beinvolved in the HIV life cycle but were not considered priority targets.

The initial discoveries of HMGA1 and BAF were not the result of a systematic search for cellular cofactors of lentiviral integration. The increasing interest in the interactomics of HIV integration and replication has resulted in algorithms for the identification and proper validation of cofactors (Figure 2). Discovery of novel HIV-1 cofactors as potential antiviral targets can be accomplished by different techniques and is often based on the search for specific and direct protein interaction partners by yeast two-hybrid (Y2H) screen or high-throughput coimmunoprecipitation (co-IP) followed by mass spectroscopy. Alternatively, full-genome RNA interference (RNAi) screens can be used to identify genes/proteins involved in HIV integration/replication.

Physical protein-protein interactions between viral protein and cofactor (Y2H and co-IP) need validation in a phenotypic assay. After specific RNAi-mediated depletion of the specific host factor, the impact on HIV replication is determined. If depletion of the candidate cofactor, verified by western blotting and RT qPCR, has no deleterious effect on HIV replication, the cofactor can be dismissed as an important cofactor of HIV replication. If depletion results in a stimulation of HIV replication, the binding partner may represent a restriction factor. In parallel, colocalization of viral protein (IN) and host protein in the cell can be verified by microscopy. Phenotypic assays measure single and multiple rounds of infection in both laboratory immortalized cell lines (e.g., HeLaP4) and primary CD4+ T cells and macrophages. In our expertise, multiple round replication represents the best assay system to validate cofactors. Use of multiple siRNAs targeting the same cofactor and back-complementation with siRNA-resistant cofactor encoding plasmids should avoid offtarget effects. Growth curves of cells depleted of cofactor should reveal major toxicity effects. An alternative method which can be also conveniently combined with RNAi to validate a cellular cofactor as a target for antiviral drug development is the use of dominant negative mutants, originally successfully exploited for interference with functions of viral proteins [46–48]. Overexpression of the integrase-binding domain (IBD) of LEDGF/p75, for example, blocks HIV-1 replication which was instrumental in studying the role of LEDGF/p75 in the HIV-1 life cycle [49, 50]. 

In case of discovery through a siRNA screen, co-IP or pull down experiments should be carried out to investigate the direct physical interaction between cofactor and viral protein. Quantitative PCR (qPCR) analysis of the different HIV DNA species (reverse transcripts, 2-LTR circles, integrants) in cells depleted for the cofactor may reveal the replication block hinting to the potential interacting viral protein (RT, IN, …). However, the expertise with siRNA screens so far has taught us that cellular pathways rather than specific PPIs are highlighted by this approach [51]. The recent efforts to use high-throughput co-IP and MS to unravel the HIV interactome should reveal more specific HIV cofactors than the siRNA screens [8].

For HIV, efficient strategies for large comprehensive Y2H screens of different cDNA libraries have been developed [52]. In the primary screen, HIV-1 IN fragments serve as baits. By combination of random and oligo-dT cDNA priming techniques, Rain et al. significantly increased the confidence of the hits by requiring identification of the same positive clone from the two independent cDNA libraries. Confirmation of the specificity of the interactions with HIV IN is done in rebound screens, where hits from the primary screen (potential cellular cofactors) are used as baits against a library of random HIV-1 protein prey fragments. This also allows mapping of respective IN binding domains [52]. By Y2H, IN interactor 1 (INI1)/hSNF5 and transportin-SR2 (TRN-SR2) were identified as IN cofactors [53, 54].

Three RNAi-based whole-genome screens for HIV infection in mammalian cells were reported in 2008 [13, 55, 56], and a meta-analysis of these studies was published in 2009 [57]. Drawbacks of these screens are the use of HeLa or HEK293T cells that are not natural host cells of HIV-1 infection. Later Thys et al. [58] demonstrated that VSV-G pseudotyping of HIV may confound interactions with natural host factors during early steps of the replication. Use of mutated or cell-line adapted viruses in the screens can be another source for false negatives and positives. The necessity of proper validation of potential cofactors derived from siRNA screens is underlined by comparison of the results of 2 large siRNA screens performed for HIV. Brass et al. [55] identified 284 genes, whereas Zhou et al. [56] picked up 232 genes. Only 15 genes overlapped between both studies [56]. LEDGF/p75 was not identified in either of them.

Nuclear import is an important step in lentiviral infection. The classical technique to study nuclear import of cellular proteins with recombinant import factors is based on digitonin-permeabilized cells [59]. The method was also adapted to study nuclear import of snRNA [60] and DNA [61]. This technique is of limited use for the study of lentiviral nuclear import since NLSs of individual viral proteins can be masked within the PIC, and the data obtained for isolated proteins do not need to fit the real situation during viral infection. There are now better approaches available for studies of lentiviral nuclear import (and early postentry steps in general) based on advances in fluorescence microscopy: real-time *in vivo* tracking [62–64] and the so-called PIC import assay [54, 65]. The PIC import assay is based on fluorescently labeled viral particles containing IN fused to eGFP (HIV-IN-eGFP) *trans*-incorporated in the particle through a fusion with HIV-1 Vpr [66].

After validation of the interaction between host factor and viral protein, drug discovery can be initiated, facilitated by high throughput screening (HTS) and high-content screening (HCS) technologies developed since the 1990s, as for example, amplified luminescent proximity homogeneous assay (AlphaScreen) technology, high-throughput FLIM for protein-protein interaction screening, enhanced chemiluminescence, fluorometric microvolume assay technology (FMAT), LeadSeeker, scintillation proximity assays (SPA), and so forth. These screening technologies allow screens to be performed efficiently, cost-effectively, and with low amounts of material. Nowadays there is a trend to move from labeled reporter assays towards label-free assays [67–69]. If structural biology approaches (crystallography, NMR, SAXS, etc.) can reveal the interface of the PPI aided by site-directed mutagenesis to corroborate the hot spots of the interaction, structure-based drug design can be embarked upon. For the discovery of LEDGINs, AlphaScreen technology and structure-based drug design were used. 

## 3. The Interaction between LEDGF/p75 and**** HIV-1 IN Is a Novel Anti-HIV Target

Today, LEDGF/p75 represents the classical example of a viral cofactor validated as druggable target for antiviral therapy. Basic academic research on the role of LEDGF/p75 in HIV infection ultimately led to development of LEDGINs, first-in-class allosteric HIV-1 integrase inhibitors [37]. 

LEDGF/p75 was originally identified in Leuven in 2002 by coimmunoprecipitation as a binding partner of HIV-1 IN [28]. LEDGF belongs to the hepatoma-derived growth factor (HDGF) family. Together with HDGF-related proteins (HRPs), this family is composed of chromatin-associated proteins. The N-terminal part of these proteins is highly conserved and contains a characteristic PWWP (Pro-Trp-Trp-Pro) domain [70, 71] (Figure 1(b)). HDGF and its homologues display between 54% and 78% sequence identity among the 91 N-terminal amino acids. Because of this similarity the amino-terminal region has been termed *Homologue to Amino Terminus of HDGF *(HATH region) [70, 71]. LEDGF/p75 is implicated in the regulation of stress response proteins. There are two splice variants of LEDGF/p75 expressed from the PSIP1 (PC4- and SFRS-interacting protein 1) gene: LEDGF/p75 and p52. They share the same N-terminal 325 amino acid residues, but have different C-termini; 205 amino acid residues in the case of p75 and 8 in the case of p52. LEDGF/p75 (530 amino acid residues) was identified as a binding partner of HIV-1 IN by immunoprecipitation of IN tetramer complexes from nuclear extracts of 293T cells expressing IN from a synthetic gene [28]. Colocalization studies with constructs of IN and LEDGF/p75 fused to GFP or HcRed1 revealed that the N-terminal and the central core domains of HIV IN are involved in the interaction with LEDGF/p75 [72]. The IN-binding domain of LEDGF/p75 was mapped to the C-terminal part of the protein and is absent from LEDGF/p52 [72]. RNAi-mediated knockdown of endogenous LEDGF/p75 expression abolished nuclear/chromosomal localization of IN [72]. This observation led to the hypothesis that LEDGF/p75 is the main chromatin-tethering factor for IN that hence determines integration site selection of *Lentivirinae* [73–75]. Through the interaction with LEDGF/p75, integration of HIV into the host cell chromatin is preferentially targeted to the body of active genes [74]. A dynamic scan-and-lock mechanism for the chromatin tethering mediated by the LEDGF/p75 PWWP domain was evidenced by a later study of Hendrix et al. [76].

Soon an evolutionary highly conserved protein-binding domain spanning amino acids 347–429 was identified by means of limited proteolysis and deletion mutagenesis [77]. This domain was coined integrase binding domain or IBD (Figure 1(b)). In the HRP family, the IBD is only present in the hepatoma-derived growth factor-related protein 2 (HRP2). In spite of the identification of the interaction between HIV-1 IN and LEDGF/p75, definition of the IBD in LEDGF/p75, a clear phenotype of IN relocalization after LEDGF/p75 knockdown, and the role of LEDGF/p75 in HIV infection remained disputed for some years, especially after one publication dismissing such role [78]. Multiple lines of increasingly solid evidence were reported in subsequent years 2005–2012 (for more extensive reviews see [7, 79, 80]). A role of LEDGF/p75 in integration and replication of HIV-1 was first suggested by the study of mutants of IN identified by Y2H screening [81]. A single mutation in IN, Q168A, disrupted the interaction with LEDGF/p75 without major effect on the catalytic activity *in vitro*. Viruses containing IN-Q168A were defective for replication and the replication block was mapped to the integration step using qPCR. Simultaneously, it was proven that LEDGF/p75 is not required for active nuclear import of the HIV PIC [81]. Using transient and stable knockdown of LEDGF/p75, Vandekerckhove et al. were first to demonstrate a close correlation between LEDGF/p75 levels and extent of HIV integration and replication [82]. Back-complementation of LEDGF/p75 restored viral replication to nearly wild-type levels [82].

In 2005, the solution structure of the IBD of LEDGF/p75 was published [83] and amino acid residues essential for the interaction with HIV-1 IN were identified: Ile365, Asp366, Phe406, and Val408. The IBD is a compact right-handed bundle composed of five *α*-helices. Residues essential for the interaction with IN are localized in the interhelical loop regions of the structure. The crystal structure of the IBD in complex with a dimeric CCD of IN was a major advance in defining the structural properties of the IBD-CCD interface [84]. The LEDGF/p75 binding pocket in IN is formed at the dimeric interface of the CCD of IN. The structure was confirmed by mutagenesis studies of Busschots et al. [85]. Two regions of the IN CCD dimer were identified to be involved in the interaction with LEDGF/p75: one centers around residues Trp131 and Trp132 while the second extends from Ile161 up to Glu170 [85].

In 2006, it was demonstrated that stable overexpression of the IBD reduces HIV replication 100-fold [49]. By competing with endogenous LEDGF/p75 for IN binding, IBD fused to eGFP was able to block HIV-1 replication at the integration step [49]. This result provided proof of concept that the HIV-1 IN/LEDGF/p75 interaction constitutes a novel target for antiviral therapy. Serial passaging of the virus in IBD overexpressing cells yielded a resistant virus with IN mutations at positions 128 and 170, located at both sides of the LEDGF/p75 binding pocket [21]. Al-Mawsawi et al. subsequently showed that a LEDGF/p75-derived oligopeptide containing the IN interacting residues Ile355 and Asp366 blocked interaction between LEDGF/p75 and IN [86]. Even though peptides and natural products have been shown to modulate PPIs in several therapeutic areas, their physicochemical properties make them less amenable for drug development [9]. Therefore, small molecule inhibitors that bind to the LEDGF/p75 binding pocket in HIV-1 IN were proposed as novel therapeutic strategy [17]. Du et al. [87] reported that a benzoic acid derivative, D77, allegedly disrupted the LEDGF/p75-IN interaction and inhibited HIV replication, albeit with cellular toxicity. Subsequently, structure-based rational drug design resulted in the identification of small molecules (CHIBA-3002 and its analogs) that reduce LEDGF/p75-IN interaction [88]. However, the first potent and selective inhibitors of HIV replication that act by disrupting LEDGF/p75-IN interaction were reported in 2010. We coined the class of small molecule inhibitors that bind to the LEDGF/p75 binding pocket in HIV-1 IN as LEDGINs. The first molecules of this class, quinolinylacetic acid derivatives, were discovered by rational drug design [37]. The reported LEDGINs have potent antiviral activity and are now in advanced preclinical development.

From the drug discovery point of view, the interactions of LEDGF/p75 with other cellular proteins are of importance. Perturbation of these interactions while targeting LEDGF/p75-IN interaction could potentially deregulate the normal cellular role of LEDGF/p75 and lead to cellular toxicity. By Y2H screens with the C-terminal domain (aa 341–507) of LEDGF/p75 as the bait, JPO2 and pogZ were identified as LEDGF/p75 binding partners and their interactions were extensively characterized [89, 90]. Maertens et al. demonstrated that interaction of JPO2 with LEDGF/p75 is mediated by LEDGF/p75 IBD, and recombinant IN competes with JPO2 for binding to LEDGF/p75 *in vitro* [91]. A positively charged patch on the surface of the IBD structure is involved in an interaction with another LEDGF/p75 binding partner, Cdc7-activator of S-phase kinase (Cdc7-ASK) [92]. LEDGF/p75 is also a crucial cofactor required for both the oncogenic and tumor suppressor functions of mixed lineage leukaemia protein (MLL)/menin complexes. MLL chimeric oncoproteins in complex with menin are dependent on the association with LEDGF/p75 [93]. Recently, the crystal structure of the ternary complex of menin-N-terminal fragment of MLL1-LEDGFIBD has been published [94].

## 4. Rational Design of LEDGF/p75-IN ****Interaction Inhibitors 

**Figure 3:**
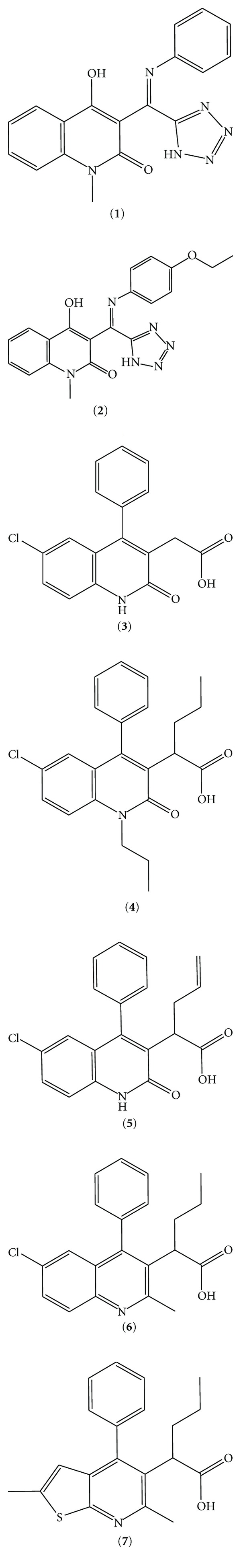
Chemical structures of the LEDGINs. Of the 25 molecules tested in AlphaScreen, compound **1** was identified as the initial hit with *in vitro* activity. Compounds **2** and **3** are commercial congeners of **1**. Compounds **4**–**7** are newly synthesized compounds with improved *in vitro* and *in vivo* activities. After serial rounds of optimization by medicinal chemistry, the early lead compounds **6** and **7** were identified with potent and selective anti-HIV activity. Compound **7** has submicromolar antiviral activity [37].

**Figure 4:**
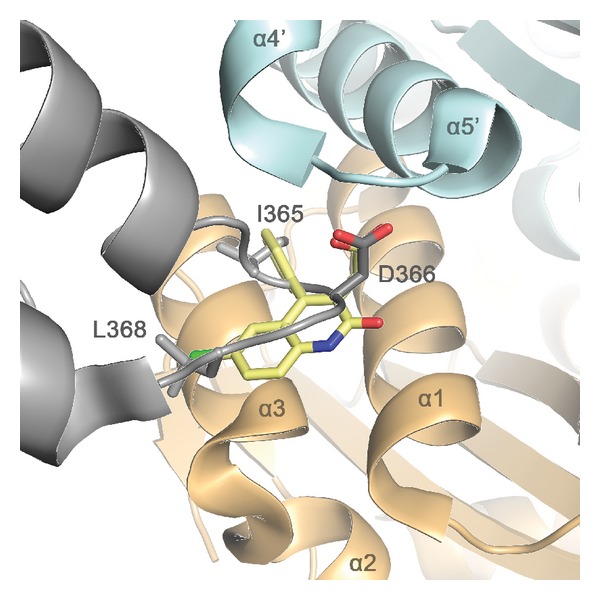
Cartoon representation of the LEDGIN **3** (yellow) superimposed with the LEDGF/p75 IBD (gray) in the pocket at the interface of the IN CCD dimer (light blue and orange). LEDGINs bind to the LEDGF/p75 binding pocket in HIV-1 IN and thereby block the interaction of the IBD of LEDGF/p75 with the dimer of the CCD, thereby interfering with tethering of the HIV-1 PIC to the host cell chromatin.

Different approaches have been employed to design and identify small-molecule inhibitors of the LEDGF/p75-IN interaction. These include large-scale screening of chemical libraries [87, 95], computational three-dimensional (3D) database screening of chemical libraries and structure-based *de novo* design [37, 88]. High-throughput screening of large libraries of chemicals against a biological target is the prevailing method for the identification of new hit compounds in modern drug discovery. Alternatively, virtual screening is based on a computer-aided survey of large libraries of chemicals that complement targets of known structure and on experimentally testing of a limited set of compounds predicted to bind well. In order to obtain *bona fide* LEDGF/p75-IN interaction inhibitors, we embarked in 2007 upon structure-based drug design [37]. Drug design is based on a virtual screen of large libraries of small molecules to fit a consensus pharmacophore docked into the region of interest. The consensus pharmacophore consists of chemical groups critical for interaction with amino acid residues or peptide backbones in the proposed drug-binding pocket. In our case the pharmacophore was designed to bind to the LEDGF/p75 binding pocket located at the interface of a dimer of the CCD of HIV-1 IN. In principle, any drug discovery project requires design, prioritization, analysis, and interpretation of results of consecutive experiments to ultimately facilitate the development of new therapeutic compounds. The rational drug design work flow used during the discovery and hit-to-lead optimization process of LEDGINs was a combination of methods. The *in silico* screen for LEDGINs integrates a multi-disciplinary approach where existing structural bioinformatics and chemoinformatics were employed in combination with a validated target-based PPI assay [37]. Different crystal structures of the HIV-1 CCD [96] and cocrystal structures with the IBD of LEDGF/p75 [84] or ligand [97] bound to the CCD were superpositioned to refine and construct more precisely a consensus pharmacophore model. Most important features in the final predictive pharmacophore model constructed for virtual screening were a “hydrophobic/aromatic” moiety overlapping with Ile365 of the IBD, a “hydrophobic/aromatic” feature overlapping with Leu368 of the IBD, “acceptor” features mimicking the acid functionality of Asp366, and a “hydrophobic/aromatic” feature overlapping with the Lys364 side chain of LEDGF/p75. 200,000 commercially available and structurally diverse compounds were filtered using the established 3D-pharmacophore query. After stringent sequential scoring and filtering of the initial libraries, 25 promising molecules with the best scoring were ordered for biological evaluation in a bead-based *in vitro* LEDGF/p75-HIV-1 IN protein-protein interaction assay in the AlphaScreen format. AlphaScreen is a bead-based medium throughput assay optimized to measure the interaction between LEDGF/p75 and HIV-1 IN [37, 89, 95]. Hits emerging from the screening were optimized by reiterative chemical refining and biological profiling in AlphaScreen and in a cell-based antiviral assay, MTT/MT4. Of the 25 molecules retained from the initial screening, four hit molecules inhibited the LEDGF/p75-HIV-1 IN interaction. One of the hit molecules, LEDGIN **1**, inhibited the PPI by 36% at 100 *μ*M and served as a starting point for structure-activity relationship (SAR) investigations aimed at the identification of more potent LEDGINs (Figure 3) [37]. Deduced SARs were used to guide synthesis of analogues with enhanced activity. The resulting early lead compounds were then further optimized in an integrated lead optimization strategy while the molecular mechanism of action was investigated in cell culture. Medicinal chemistry optimization, aided by structural information provided by high-resolution cocrystals of LEDGIN **3** soaked into the HIV-1 CCD (Figure 4), generated congeners of LEDGIN **3** (including LEDGIN **6** and **7**) with improved biological activity (Figure 3).

Furthermore, LEDGINs did not interfere with the interaction between LEDGF/p75 and its cellular binding partners JPO2 or pogZ, conforming their specificity. Of note, Hou et al. [95] identified several compounds inhibiting the LEDGF/p75-IN interaction through high-throughput screening of a compound library of more than 700,000 small molecules with AlphaScreen. However, the quinolinylacetic acid derivatives are the first examples of potent and specific inhibitors of HIV-1 replication which have been extensively evaluated for their therapeutic potential and mechanism of action in cell-based antiviral assays (including in primary cells) [37].

## 5. LEDGINs as Therapeutics

A critical evaluation of the mechanism of action and therapeutic potential of LEDGINs requires investigation of different drug characteristics: (a) a high binding affinity and specificity to HIV-1 IN, (b) potent and broad spectrum anti-HIV activities in cell-based antiviral assays, (c) lack of toxicity, and (d) a optimal pharmacokinetic (PK) and pharmacodynamic (PD) profile allowing a once a day administration in patients. We could demonstrate that inhibition of the LEDGF/p75-HIV-1 IN interaction by LEDGINs blocks HIV integration [37]. Integration inhibitors are characterized by a typical pattern of viral DNA species as measured by qPCR. 2-LTR circles are the dead-end byproduct of nonintegrated viral DNA; their number is increased upon integration block if upstream steps are not hampered [98]. We showed that both the classical integrase strand transfer inhibitor (INSTI) raltegravir and LEDGINs reduce the number of integrated proviral DNA and increase the number of 2-LTR circles without effect on reverse transcription. Resistance selection in cell culture against a new class of antiviral agents ultimately corroborates the antiviral target. By serial passaging of HIV-1 in increasing concentrations of LEDGIN **6**, we selected a resistant strain with the A128T substitution in IN. The A128 residue is a hot spot of the IN-LEDGF/p75 interface and was included in the predictive pharmacophore model for the virtual screen. The resistance mutation, thus, corroborates the specificity of LEDGINs. The A128T mutation in integrase is not associated with resistance to INSTIs and LEDGINs lack cross-resistance with other ARV classes corroborating their novel mode of action. Of note, it was recently shown that LEDGINs can also block the interaction between HRP-2 and HIV IN in the absence of LEDGF/p75 [99].

In conclusion, there are obvious advantages of drugs targeting LEDGF/p75-IN interaction. LEDGINs show a pathway of resistance development that is different from that of the INSTIs and lacks cross-resistance with ARV in the clinic [100]. Discovery of LEDGINs is a good example of structure-based rational drug design targeting a well-defined and biologically relevant PPI.

## 6. HIV Integrase Cofactors and Nuclear Import

To accomplish their life cycle, retroviruses need to integrate their genetic material into the host DNA in the nucleus. For this purpose, retroviruses developed distinct strategies to overcome the nuclear membrane barrier. Gammaretroviruses such as murine leukemia virus (MLV), for example, cannot pass nuclear pore complexes and only integrate during mitosis after breakdown of the nuclear membrane [101]. Lentiviruses such as HIV in contrast are able to infect both dividing and nondividing cells [102, 103]. Many factors, both from viral and host cell origin, have been suggested to take part in the nuclear import of the lentiviral preintegration complex (PIC) (for reviews see [104, 105]). Nuclear import is a bottleneck in lentiviral infection, and cellular cofactors of this process are attractive targets for anti-HIV therapy. Although recent studies shed light on lentiviral PIC transport to the nucleus, general consensus on the importance of particular viral and cellular players still has to be established. From the viral proteins present in the PIC IN, matrix (MA) and viral protein R (Vpr) were suggested to affect nuclear import, and several nuclear localization signals (NLSs) were identified in these proteins (for review see [105]). A *cis-*acting central polypurine tract (cPPT), a sequence present almost exclusively in the lentivirus genus and used for initiation of plus-strand synthesis, may as well affect the efficiency of nuclear import [106–108]. However, HIV with mutations in each of the NLSs still remained infectious in nondividing cells [109]. Yamashita and Emerman, using HIV chimeric viruses in which the entire IN sequence was replaced by that of MLV, and all the other NLSs in MA, Vpr, and cPPT were eliminated, demonstrated that neither of these NLSs is essential for the ability of HIV to infect nondividing cells [110]. Despite the fact that none of the above-mentioned viral elements appears absolutely required for nuclear import, a major effect of the cPPT on the kinetics of viral DNA entry into the nucleus was demonstrated [108, 111, 112]. After excluding a role for the previously reported viral NLSs in lentiviral nuclear import, two major explanations for the cell cycle independence of lentiviral nuclear entry prevail. Limited uncoating of the gammaretroviral capsid may interfere with importin-mediated transport through the nucleopore, whereas timely disassembly of the lentiviral capsid may allow interaction with importin(s). Alternatively, interaction with components of the nuclear import machinery may be restricted to proteins present in the lentiviral PIC. For a discussion on the impact of the lentiviral capsid on nuclear import, we refer to [113–115]. 

Several nuclear import factors and nucleoporins (Nups) have been implicated in HIV nuclear import: importin *α*1* [116–119], importin *α*3* [120, 121], importin 7* [122, 123], Nup153* [13, 55, 124–127], Nup62* [128], Nup54 [129], Nup85 [55, 125], Nup98 [13, 130–132], Nup107 [55, 125], Nup133 [55, 125], Nup155 [130], Nup160 [55, 125], Nup210 [130], Nup214 [13], and Nup358/RanBP2 [13, 55, 115, 125, 133, 134] (proteins with * were shown to interact with IN).

Importin *α*1/Rch1 was the first karyopherin shown to interact with HIV-1 IN [117]. The study was initiated by the observation that the growth defect of a HIV-1 MA/Vpr double deletion mutant in terminally differentiated macrophages was masked at high MOI. These data pointed to an activity that can substitute for MA and Vpr in the nuclear import of the HIV-1 PIC. Authors showed that HIV-1 IN is a karyophilic protein, detected IN-Imp*α*1 interaction, and defined two NLSs (one around positions 186–189 (KRK^188^) and one encompassing residues 211–219 (KELQKQITK^219^)) in the C-terminal region of HIV-1 IN as responsible for the interaction [117]. The IN-Imp*α*1 interaction was initially confirmed by *in vitro* binding studies [119, 135], but questioned later by work of Ao et al. [123]. The Imp*α* family contains 6 isoforms grouped into 3 subfamilies with a primary sequence identity between 50 and 85% [136]. *In vitro* studies suggest that various isoforms can recognize the same NLS-containing proteins, although with different binding efficiency [120]. Therefore, Ao et al. [120] investigated the contributions of the different Imp*α* isoforms to HIV-1 replication. Via shRNA, mediated knockdown Imp*α*3 was shown to be required for efficient HIV infection of HeLaP4 cells, T cells, and primary macrophages. qPCR analysis revealed that Imp*α*3-knockdown resulted in a significantly reduced level of 2-LTR circles, suggesting a role in HIV nuclear import. By immunoprecipitation, the HIV-1 IN-Imp*α*3 interaction was attributed to the C-terminal domain (CTD aa 250–270) of IN. Imp*α*1 and Imp*α*5 also affected HIV infection [120]. The importance of importin *α* isoforms for HIV nuclear import was questioned by work of Depienne et al. [137] who studied nuclear import in digitonin-permeabilized HeLa cells. According to these authors, nuclear accumulation of IN (as a protein) does not involve karyopherins *α*, *β*1, and *β*2-mediated pathways and is also independent of GTP hydrolysis and Ran [137]. Here, we raise again the question whether nuclear import of IN is relevant for the nuclear entry of the HIV PIC. Importin 7 (Imp7) has also been implicated in HIV-1 nuclear import. Originally, it was proposed as a HIV-1 nuclear import factor by Fassati et al. based on nuclear import of purified HIV-1 reverse transcription complexes in digitonin-permeabilized HeLa cells and primary human macrophages [122]. However, when Zielske and Stevenson depleted Imp7 by 80–95% in primary macrophages and HeLa cells using RNAi, neither the rate nor the extent of HIV-1 or SIV cDNA synthesis or nuclear translocation was affected [138]. In a direct comparison using coimmunoprecipitation, HIV-1 IN was found to interact with Imp7, but not with Imp*α*1/Rch1 [123]. Finally, the Fassati group admitted that Imp7 is not essential for HIV-1 infection but maximizes nuclear import [139]. 

In a full-genome siRNA screen, Nup153, Nup214 and Nup358 were found to play a role in the nuclear import and Nup98 in the integration of HIV [13], although detailed validation still had to be performed. Nup153 and Nup358/RanBP2 are the most extensively studied Nups in the context of HIV infection. Nup153 has been shown to interact with HIV-1 IN, and the interaction is mediated by its C-terminal domain rich in FxFG repeats[124]. When added in excess to the semipermeabilized import assay, the C-terminal domain of Nup153 inhibited the nuclear import of HIV-1 IN [124]. Interestingly, codepletion of Nup153 and transportin-SR2 (TRN-SR2) yielded synergistic effects, that outweighed those calculated based on individual knockdowns, indicating potential interdependent roles for these factors during HIV-1 infection [127]. Nups requirement for HIV-1 infection was further studied by Lee et al. [125]. HIV-1 infection was impaired by Nup358/RanBP2, Nup153, or Nup160 knockdown. In contrast, infection by the HIV-1 CA N74D mutant (see below) was less dependent on Nup358/RanBP2 and Nup153, suggesting that these proteins interact, directly or indirectly, with CA during infection [125].

Nup62 has been shown to act at several steps during HIV-1 replication. Monette et al. first showed that HIV-1 replication markedly alters the localization of Nup62 and that its expression is linked to the nuclear export of the unspliced viral genomic RNA [140]. Later proteomics and immunogold electron microscopy studies showed that HIV-1 infection induces extensive changes in the composition of the nuclear envelope and its associated proteins and identified Nup62 as a component of purified virus [141]. Former observation is particularly important for consideration of the involvement of individual Nups and Nups-interacting partners (like importins) in HIV infection. HIV-1 can via remodeling of the nuclear pore complexes (NPCs) make accessible Nups which facilitate nuclear import and/or integration, and the process of remodeling can have impact not only on late stages of infection (production of the progeny), but also on the mentioned early steps. Encapsidated Nup62 may be required for efficient nuclear import of the PIC in newly infected cells [141].

Nup62 has recently been proposed as a binding partner of HIV-1 IN [128]. GST-tagged IN was able to pull down Nup62. The specificity of the interaction was further proven by co-IPs. Nup62 knockdown in CD4+ T cells and macrophages significantly inhibited HIV-1 infection and by qPCR analysis, the block of the infection was pinpointed to viral integration and in a much lesser extent to the nuclear import step. Subcellular protein fractionation showed that Nup62 binds to chromatin, interacts with HIV-1 IN both in the nuclear and chromatin bound extracts, and knockdown of Nup62 significantly reduced the association of the IN with chromatin causing impaired HIV-1 integration observed also by qPCR. Finally, expression of the C-terminal domain of Nup62 in CD4+ T cells reduced the association of IN with chromatin and did inhibit HIV-1 infection [128].

HIV integration is favored in chromosomal regions rich in active transcription units and associated features such as CpG islands, DNAaseI hypersensitive sites, and high G/C content [142]. Integration site sequencing offers a new view on how HIV-1 uses the host nuclear import machinery to reach its integration sites [115, 134]. Wild-type HIV-1 in the presence of cyclosporine (Cs), HIV-1 CA mutants deficient for CypA interaction (CA G89V or P90A), and chimeric HIV-1 containing SIVmac CA, all integrate in genomic areas of high gene density/activity. On the contrary, HIV-1 capsid mutants that are less sensitive to TRN-SR2, Nup358 or Nup153 depletion by RNAi (CA N74D or N57A) integrate in genomic areas of low gene density/activity. Both groups of CA mutants were impaired in replication in HeLa cells and human macrophages. In accord with the observed differences in integration pattern, a block of engagement of CypA/Nup358 by mutating the virus CA or by inhibiting cellular CypA with cyclosporine force HIV-1 to use for nuclear import and integration a Nup358/Nup153-independent pathway [115].

In 2008, transportin-SR2 (TRN-SR2, TNPO3) was independently identified as a cellular cofactor of HIV-1 replication in two siRNA screens [13, 55] and as a HIV-1 IN binding partner by Y2H screening [54]. Although its exact role in HIV-1 infection has not been fully clarified, several independent studies confirmed TRN-SR2 as a genuine cellular cofactor to the extent that it is now being used as a positive control in HIV-1 interaction studies [143]. 

## 7. Transportin-SR2 as a Cofactor of HIV Nuclear Import

**Figure 5:**
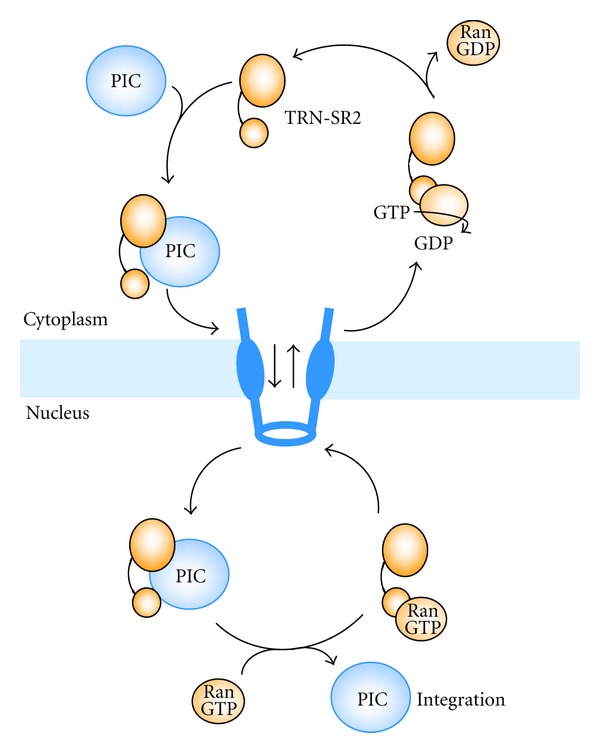
Scheme of nuclear import of the PIC and TRN-SR2 recycling.

TRN-SR2 belongs to the importin-*β* superfamily of karyopherins [144]. The protein has 975 amino acid residues and is composed of *α*-helical HEAT repeats. TRN-SR2 is known to import essential splicing factors, the serine/arginine-rich proteins (SR proteins), to the nucleus and is, therefore, involved in the regulation of both constitutive and regulated precursor mRNA splicing. The recognition of the SR-proteins by TRN-SR2 relies on the conserved RS-domain and requires phosphorylation [144–146] although TRN-SR2 is known to import as well proteins not belonging to the SR protein family [147, 148]. The RS domain of SR proteins serves both as an NLS and a subnuclear localization signal [149, 150]. A TRN-SR2 mutant deficient in Ran binding colocalized with SR proteins in nuclear speckles [146]. TRN-SR2 binds its cargo in the cytoplasm and via its interaction with the nuclear pore proteins translocates with cargo to the nucleus (Figure 5). The import is linked to the RanGTP/RanGDP cycle. The small protein Ran GTPase is a member of the Ras protein superfamily and the motor of nuclear protein import. Interaction between Ran and karyopherins is modulated by the state of the bound nucleotide (GTP or GDP). In the nucleus, RanGTP binds to TRN-SR2, displaces the cargo, and then shuttles together with TRN-SR2 to the cytoplasm, where GTP is hydrolyzed to GDP. In the GDP-bound state, Ran dissociates from TRN-SR2 enabling a new round of nuclear import [151]. TRN-SR2 has been shown to interact with Nup62 or its associated complex [146]. Of note, Nup62 is translocated to the cytoplasm and encapsidated into HIV-1 virions during HIV-1 infection [140, 141].

ASF/SF2 has been proven to affect the splicing pattern of HIV RNA transcripts [152, 153]. The nuclear import of this splicing factor is mediated by TRN-SR2 and this was the first indication of a possible involvement of TRN-SR2, in HIV replication. TRN-SR2 was identified as a cellular cofactor of HIV-1 in the RNAi genome-wide screens [13, 55], but not in the Zhou screen [56]. TRN-SR2 knockdown had little or no effect on murine leukemia virus (MLV) transduction [54, 55]. Interaction of TRN-SR2 with HIV-1 IN was originally detected in a Y2H screen of a random primed CEMC7 cDNA library with HIV-YU2 IN as bait [54]. Exclusivity of the interaction with viral IN was verified in a reverse screen against a library of HIV genome DNA fragments. The specificity of the interaction of HIV-1 IN with TRN-SR2 was confirmed in pulldown assays [54]. SiRNA-mediated knockdown of TRN-SR2 resulted in a 6-fold inhibition of HIV replication in HeLaP4 cells [54]. TRN-SR2 specific shRNA reduced infectivity of both HIV-1 (~8- to 10-fold) and SIVmac (~20-fold) [54, 115]. Interestingly, IN inhibitor-resistant viruses are still susceptible to TRN-SR2 knockdown [54]. By real-time qPCR, the block in HIV replication was mapped to a moment after reverse transcription and prior to integration, which coincides with nuclear import [54]. The import assay with IN-eGFP labeled virus [65] was used to corroborate the role of TRN-SR2 in HIV nuclear import [54]. After depletion of TRN-SR2 using red fluorescent siRNA, the treated cells were infected by HIV-IN-eGFP. In cells positive for the red fluorescent label, the numbers of PICs present in the nucleus versus the cytoplasm were counted. The nuclear/cytoplasmic ratio of PICs dropped 5-fold in the TRN-SR2 depleted cells [54].

A possible role of lentiviral capsid in TRN-SR2-mediated nuclear import was suggested by the finding that both a chimeric HIV virus, carrying MLV capsid (CA), MA and p12 proteins, and a HIV-1 strain, carrying the CA N74D mutant, apparently were insensitive to TRN-SR2 knockdown [58, 125, 154, 155]. Authors concluded that the viral capsid and not IN determines TRN-SR2 dependency of HIV infection. One should be careful with interpretation of some data. Some studies [154, 156] were done with pseudotyped HIV virus carrying the vesicular stomatitis virus G envelope (VSV-G), known to induce receptor-mediated endocytosis instead of membrane fusion as a way to enter the cell. Moreover, VSV-G pseudotyped HIV does not engage chemokine coreceptors (CCR5, CXCR4) known to induce signal transduction cascades in the cell [157]. When TRN-SR2 knockdown cells were infected with viruses carrying the wild type HIV-1 envelope, the HIV-1 N74D CA mutant regained sensitivity to TRN-SR2 knockdown [58].

TRN-SR2 is not used to the same extent as a nuclear import factor by all lentiviruses [58, 154, 156] but a direct correlation between the phenotype in cell culture and the *in vitro* PPI has not yet been documented. Logue et al. showed that the *Drosophila* TRN-SR2 can substitute for its human counterpart and defined the cargo-binding domain of TRN-SR2 as required for lentivirus infection [156]. From the IN part of the interaction, IN mutations previously characterized to impair LEDGF/p75 binding (W131A, Q168L) were insufficient to affect nuclear import [158]. Zhou et al. recently proposed a model in which CA along with tRNAs is export cargoes for TRN-SR2 in a RanGTP-dependent way [159]. According to this hypothesis, TRN-SR2 modulates nuclear uncoating of imported PICs by removing any remaining CA proteins and tRNAs blocking the integration step and promotes nuclear export of these viral components. The model suggests that efficient HIV-1 integration depends on this TRN-SR2 activity [159]. Another study hinted at a role for TRN-SR2 prior to integration. HIV integration site selection was modified by depletion of TRN-SR2 and Nup358/RanBP2 [134]. However, this observation can alternatively be explained by the fact that correct trafficking through the NPC may facilitate the subsequent integration step. Although a clear understanding of HIV nuclear import and on the role of TRN-SR2 requires more experimentation, all data are consistent with a close link between HIV uncoating in the cytoplasm and nuclear import on the one hand, and nuclear import and integration on the other hand.

## 8. Conclusions

This paper highlights the importance of research on cellular cofactors of HIV replication as potential targets for anti-HIV drugs. The interaction between LEDGF/p75 and IN is crucial for HIV replication, and the rational design of LEDGINs as novel antivirals represents an important achievement in translational research. Efficient targeting of host-virus PPIs expands the possible arsenal of targets beyond HIV-encoded enzymes. This novel paradigm can be extended to other viral diseases. Increased understanding of the virus-host interactome can be the basis for plenty of future antivirals. Since PPIs have pivotal roles in virtually all physiological and disease-related intracellular macromolecular complexes, development of SMIPPIs can benefit many therapeutic areas. While the example described here is particularly relevant to the field of virology, applications of SMIPPI technology to other fields will increase as our knowledge on the role of PPIs in human diseases expands. 

Since the nuclear import of PICs still represents a black box in our knowledge of HIV infection and since IN plays an active role at this stage, study of the IN interactome may also shed light on this process. The discovery that the importin TRN-SR2 is a binding partner of IN can provide the lever to open this box. Research on HIV nuclear import not only provides us with insights in basic virology, but also has great potential for drug discovery especially since nuclear import is a bottleneck in HIV replication. There is increasing evidence that lentiviral chromosomal target site selection for integration is linked to nuclear import of PICs. Moreover, proper illumination of the lentiviral route to the nucleus and of the impact on integration site selection will aid the design of safer gene therapy approaches.
